# Technology-Mediated Communication in Familial Relationships: Moderated-Mediation Models of Isolation and Loneliness

**DOI:** 10.1093/geront/gnaa040

**Published:** 2020-05-05

**Authors:** Vanessa Burholt, Gill Windle, Merryn Gott, Deborah Jane Morgan

**Affiliations:** 1 School of Nursing, Faculty of Medical and Health Sciences, University of Auckland, New Zealand; 2 School of Population Health, Faculty of Medical and Health Sciences, University of Auckland, New Zealand; 3 Centre for Innovative Ageing, College of Human and Health Sciences, Swansea University, Wales, UK; 4 School of Health Sciences, Bangor University, Wales, UK

**Keywords:** Telephone, Computer-mediated communication, Social relationships, Families, CFAS Wales study

## Abstract

**Background and Objectives:**

We examined whether technology-mediated communication has functional or emotional equivalence to face-to-face (FtF) contact in familial relationships, by scrutinizing the effects of phone, text/e-mail, and video contact on isolation and loneliness.

**Research Design and Methods:**

We tested whether FtF contact with a relative would mediate the pathway between proximity to family and (i) isolation and (ii) loneliness. We then tested hypotheses that telephone, text/e-mails, and video contact would moderate this mediated pathway. We compared models for younger (<75) and older (≥75) cohorts, expecting to observe moderation effects for text/e-mail and video contact in the younger cohort only. Data were drawn from Wave 2 of CFAS Wales (United Kingdom) study (*N* = 2,099).

**Results:**

Proximity to a relative had a significant indirect effect on isolation and loneliness through the mediating variable FtF contact. Phone and text/e-mail contact moderated the effect of FtF contact on isolation for all samples. None of the technologies moderated the impact of FtF contact on loneliness for the full sample. Telephone contact had a moderating influence on loneliness for the younger cohort only. Video calls had no significant moderation effect.

**Discussion and Implications:**

Telephone and text/e-mail contact have functional equivalence to FtF contact in familial relationships. None of the forms of technological communication have emotional equivalence to the “gold standard” of embodied presence. The study demonstrates the importance of theorizing about the pathways to isolation and loneliness to better understand the likelihood of implementing successful interventions using technology-mediated communication within families.

## Background

Social isolation has been associated with adverse outcomes such as poor health (Shankar, McMunn, Demakakos, Hamer, & Steptoe, 2017), mortality (Holt-Lunstad, Smith, Baker, Harris, & Stephenson, 2015), reduced wellbeing (Golden et al., 2009), and loneliness (e.g., Burholt, Windle, & Morgan, 2016; Burholt et al., 2019). Similarly, an extensive body of research evidence has associated loneliness with negative consequences such as mortality (e.g., Holt-Lunstad et al., 2015), dementia (e.g., Holwerda et al., 2014), and poor functional status (e.g., Shankar et al., 2017). In the 1960s, research suggested that changes in family structure and population mobility contributed to the risks for social isolation and loneliness for older people (Townsend, 1969). Recently, the World Health Organization has suggested that transnational migration, decreasing fertility rates, and shrinking family sizes are likely to increase social isolation globally (World Health Organization, 2015). Despite some evidence to the contrary (Burholt & Sardani, 2017; Victor et al., 2002), this notion persists and is fuelled by media representations of societal and/or familial failures underpinning the risks for loneliness and isolation in the older population (Agren, 2017; Breheny & Severinsen, 2018).

Technology-mediated communication can bridge the physical distance between kin, staving off social isolation, and loneliness (e.g., Cutler, 2015). Indeed, some academics pronounced the technological revolution as “the death of distance” (O’Brien, 1992) and “the end of geography” (Cairncross, 1997). However, there is a growing body of evidence that technology-mediated communication can displace face-to-face (FtF) contact and have a negative impact on social networks, increasing isolation and loneliness (Kraut et al., 1998). Despite an ideological dissonance between the utopian and dystopian views of the effects of technology-mediated communication on social life, very few robust studies have examined the everyday communication practices of older people and their impact on social isolation and loneliness.

### Theoretical Underpinnings

**Figure 1.**
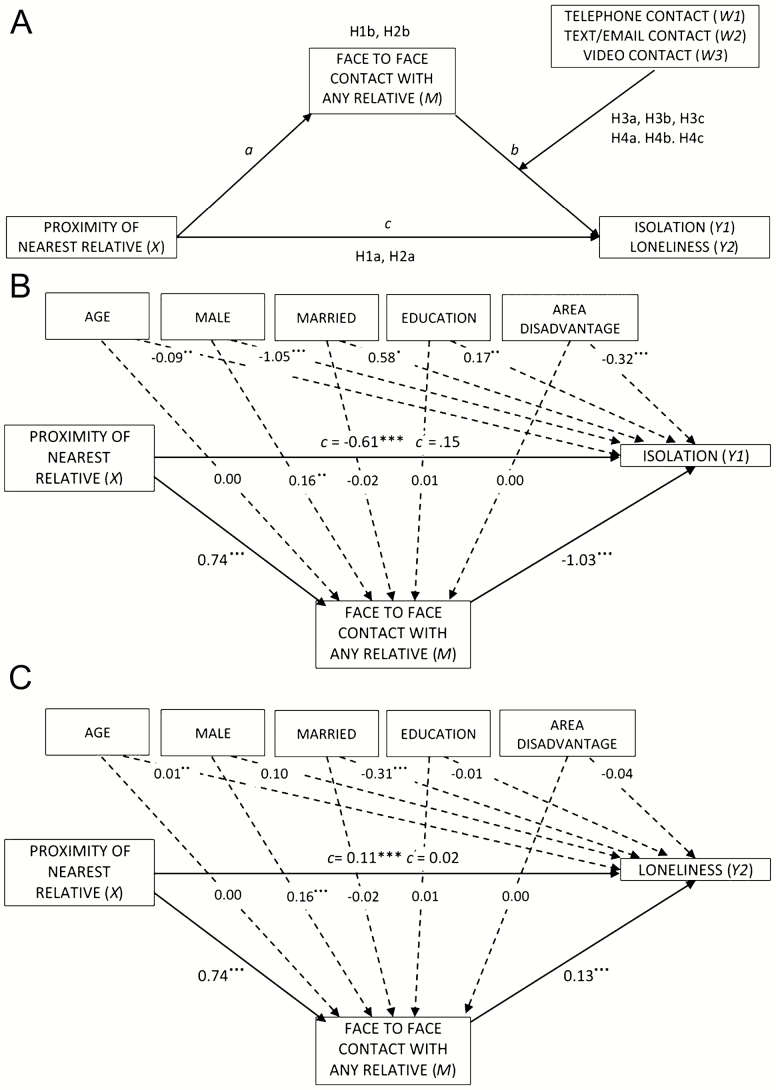
Hypothesized model (A) and statistical mediation models indicating the beta coefficients for proximity of relative (*X*), face-to-face contact (*M*), controls and social isolation (*Y1*) (B) and loneliness (*Y2*) (C) for the total sample (*N* = 2,099).

Social relations can be operationalized by assessing meaningful social contact or network size: Low levels of both may be referred to as social isolation (Lubben et al., 2006). Geographically dispersed families require considerable resource investment to maintain physical FtF contact over greater distances. Building on this premise, we predict that closer proximity to at least one relative will be associated with less social isolation (H1a), and this association will be mediated by FtF contact (H1b) (Figure 1A).

We define loneliness as a negative emotional experience that is the reaction to a mismatch between expectations of the quality and quantity of social relationships and those that are achieved (Perlman & Peplau, 1981). There are differences between individualistic and collectivist cultures in the “ideal” mix of family and friends that protect against loneliness (Burholt, Dobbs, & Victor, 2017; Morgan et al., 2019). However, the centrality of family relationships for older people, especially the parent–child relationship, is acknowledged. Relatives who live in close proximity and who are able to provide contact reduce the risk of loneliness for older family members (De Jong Gierveld, Van Tilburg, & Dykstra, 2018). Consequently, we predict that closer proximity to at least one relative will be associated with less loneliness (H2a), and this association will be mediated by FtF contact (H2b) (Figure 1A).

### Functional Equivalence of Technology-Mediated Communication to FtF Contact

In order to assess the impact of technology-mediated communication on social isolation in the face of geographical separation between family members, we draw on the functional equivalence perspective. According to this position, “a new technology will replace those activities that most closely perform the same functions for the users as did the older technologies” (Robinson & De Haan, 2006, p. 2).

The telephone has been a feature of UK households for around half a century. The percentage of households in the United Kingdom with landlines increased from 35% in 1970 to 81% in 1985 and peaked at 95% in 1998–2000 before declining, as sales of mobile phones increased (Office for National Statistics, 2019). At this time, other forms of technology-mediated communication were mainly text or e-mail and accessed through mobile phone providers, mailer systems, or internet service providers (Herring, 2004). In the last few years, the development of the internet, increased bandwidth, and faster connections for home users has meant that multimedia applications incorporating audio and video have become more accessible. In 2018, in the United Kingdom, 92% of people aged 62–74 years and 81% of people aged 75+ years owned a mobile phone, but fewer (47% and 26%, respectively) used a smart phone with the capacity for multimedia applications (Rossiter-Base, 2019). Although new communication products have replaced older ones, functional equivalence can only be estimated by examining social practices and their impact on outcomes.

Assumptions about the functional equivalence of technology-mediated communication to FtF contact for older people have led to a proliferation of studies focusing on *how* grandparents keep in touch with adult children and grandchildren living at a distance (e.g., Busch, 2018; King-O’Rian, 2015) and/or transnational families stay connected (e.g., Burholt, 2004; Burholt, Dobbs, & Victor, 2016). These studies focus on the instrumental process of communication and contact, but do not consider the outcomes of the process. In the present study, we take a “utopian” perspective that three types of technology-mediated communication (synchronous audio telephone calls; asynchronous written texts/e-mails; and synchronous audiovisual video calls, e.g., Skype, FaceTime, and Google Hangouts) will supplement or provide a substitute for FtF contact rather than displacing it. We hypothesize that technology-mediated communication will have some functional equivalence with FtF contact. The negative relationship between more limited FtF contact and greater isolation will be diminished by (moderated-mediation model) telephone contact (H3a), text/e-mail contact (H3b), and video contact (H3c) (Figure 1A).

### Emotional Equivalence of Technology-Mediated Communication to FtF Contact

The functional equivalence approach does not consider how different technologies fit with long-term psychological goals. Although technology-mediated communication may be instrumental in achieving social contact, it could be argued that communication is a stepping stone to the long-term goal of avoiding feelings of loneliness. Scientific articles that have explored the impact of using technology-mediated communication on the reduction of loneliness have tended to report on intervention studies that deliver therapies (e.g., cognitive-based stimulation therapy; Dodge et al., 2015), provide social support (e.g., Evans & Jaureguy, 1982), befriending (e.g., Cattan, Kime, & Bagnall, 2011), or friendship groups (e.g., Mountain et al., 2014), or improve opportunities for social connectiveness (e.g., Shapira et al., 2007). Very few studies have examined the emotional equivalence of using different forms of communication to supplement, or as a substitute for FtF contact between family members.

Noninterventional studies have shown that lower loneliness scores were significantly related to the daily number of incoming phone calls for older adults in the United States (Petersen, Thielke, Austin, & Kaye, 2016), and technology-mediated communication between older internet users living in Australia and their relatives and friends (Sum, Mathews, Hughes, & Campbell, 2008). However, both studies were relatively small (*N* = 26 and *N* = 222, respectively) and neither controlled for FtF contact. In the present study, we examine whether technology-mediated communication protects against loneliness. According to our definition of loneliness (Perlman & Peplau, 1981), technology-mediated communication will decrease loneliness if these forms of contact meet the expectations for familial relationships held by older people. We will interpret a positive impact on loneliness as emotional equivalence to FtF contact. Therefore, we hypothesize that technology-mediated communication will have some emotional equivalence with FtF contact. The negative relationship between more limited FtF contact and greater loneliness will be diminished by (moderated-mediation model) telephone contact (H4a), text/e-mail contact (H4b), and video contact (H4c) (Figure 1A).

### Cohort Differences in Functional and Emotional Equivalence of Technology-Mediated Communication to FtF Contact

The choice between technologies and the likelihood that they will be used to supplement or as a substitute for FtF contact (i.e., have functional equivalence to FtF contact) is likely to depend on the resources (e.g., competencies, access to technology-mediated communication) and preferences of the older adult. While the telephone is commonly used for long-distance communication by older people (Moffatt, David, & Baecker, 2013), other types of technology-mediated communication may require digital skills that vary across the population (Robinson & De Haan, 2006). For example, younger cohorts of older people are more likely than older cohorts to be computer users as they may have learned to use them in the workplace (Carpenter & Buday, 2007). Building on the previous analysis, we will examine the significant moderated-mediation models by cohort (<75 years; ≥75 years) and predict that there will be significant moderated mediation with social isolation as an outcome and phone contact as a moderator for both cohorts (H5a); text/e-mail contact (H5b) and video contact (H5c) as moderators for the younger cohort only.

In terms of “expectations” about the use of technology-mediated communication to meet social needs and have emotional equivalence with FtF contact, it would follow that the telephone would be more familiar to older old people than texting or e-mailing, thus more likely to meet expectations for how relationships may be maintained. Video calls would be the least familiar. We would expect there to be differences in pathways to avoiding loneliness between younger and older cohorts. We predict that there will be a significant moderated-mediation model with loneliness as an outcome and phone contact as a moderator for both cohorts (H6a); text/e-mail contact (H6b) and video contact (H6c) as moderators for the younger cohort only.

## Design and Methods

### Sample and Procedure

Cross-sectional data are drawn from Wave 2 of the Cognitive Function and Ageing Study (CFAS Wales), a nationally representative study of community-dwelling people aged 65 and older in Wales, UK. In Wave 1 (2012–2014), participants were randomly sampled from primary care registration lists in three Local Authorities in Wales: Neath Port Talbot, Gwynedd, and Anglesey. Sampling was stratified according to age group (65–74 years: ≥75 years). Three thousand five hundred ninety-three computer-assisted personal interviews were conducted in English or Welsh in participants’ homes (Burholt, Windle, et al., 2016). In Wave 2, a follow-up interview was conducted with 2,236 participants (62.2% of the Wave 1 sample) approximately 2 years after the baseline interview (2014–2016). Of the original sample, 195 (5.4%) had died, and 1,162 (32.3%) were lost between waves. The response rate in those still alive and contactable at Wave 2 was 70.6%. This article is based on a sample of 2,099 participants from Wave 2 with living relatives, and no missing data on the variables used in the analysis.

### Measures

#### Independent Variable

Proximity of nearest relative (not spouse) (X) was ascertained by asking participants “How far away, in distance, does your nearest child or other relative live?” Ordinal responses categories were same house or within 1 mile (1), 1–5 miles (2), 6–15 miles (3), 16–50 miles (4), and 50+ miles (5).

#### Dependent Variables

Social isolation (Y1) was measured using the six-item Lubben Social Network Scale (LSNS-6). The questions evaluate the frequency of contact and quality of kin and nonkin relationships. Score ranges from 0 (high isolation/few social resources) to 30 (low isolation/many social resources). The six-item scale has a reported alpha coefficient of 0.8 (Lubben et al., 2006) and in the present study was 0.74 at Wave 1 and 0.73 at Wave 2. A score of less than 12 is used as a clinical cut point to indicate social isolation (Lubben et al., 2006).

Loneliness (Y2) was measured using the six-item De Jong Gierveld scale. The score is the sum of all items, where higher scores represent greater loneliness. The six-item scale has a reported alpha coefficient of reliability ranging from 0.70 to 0.76 (De Jong Gierveld & Van Tilburg, 2006). Although reliability in Wave 1 of the study was 0.77, it was only 0.56 in Wave 2 indicating greater homogeneity in loneliness scores in the follow-up sample. A score in the range of 2–6 on the loneliness scale was used to identify participants that were lonely (De Jong Gierveld & Van Tilburg, 2006).

#### Mediating and Moderating Variables

Frequency of FtF contact (M) with a relative (not spouse) was ascertained by asking participants “How often do you see any of your children or other relatives to speak to?” Interviewers were instructed to ascertain cumulative contact (i.e., if the person saw a different relative every day this would be rated as daily) and in-person FtF contact not using technology-mediated communication. The same question was repeated and reworded to ascertain frequency of phone contact (W1), text, or e-mail contact (W2), and video contact (W3), for example, “How often do you speak to your children or other relatives over the phone?” Ordinal responses categories were daily (1), two to three times a week (2), at least weekly (3), at least monthly (4), and less often (5).

#### Covariates

Demographic covariates (C_1_–C_5_) used in the analysis were self-reports of age (years) (C_1_), gender coded as male (1) or female (0) (C_2_), married (1) or not (0) (C_3_), and full-time education (years) (C_4_). To control for access to technology-mediated communication, area deprivation was also included as a covariate. This operationalized using the Welsh Index of Multiple Deprivation (WIMD) 2014, which is the official measure of relative deprivation for small areas in Wales (Welsh Government, 2015). WIMD comprises eight standardized domains of deprivation: income, employment, health, education, access to services, community safety, physical environment, and housing. A score is calculated for Lower Super Output Areas (LSOAs; a geographical locale which contains on average 1,600 individuals). Each of the 1,909 LSOAs in Wales are ranked according to the level of deprivation indicated by domain and aggregated domain scores. Analysis used quintiles of the aggregated WIMD from most deprived (1) to least deprived (5) (C_5_).

### Analytical Procedure

Descriptive statistics were produced for all variables (Supplementary Table 1) for the sample (*N* = 2,099) and group comparisons were made between younger (<75 years; *n* = 1,051) and older (≥75 years; *n* = 1,048) cohorts. Correlation analysis examined covariation between all variables in the model (Supplementary Table 2). A variance inflation factor (VIF) was calculated for each predictor in the models, with values greater than 10 indicating a high degree of multicollinearity (Hair, Anderson, Tatham, & Black, 1995).

Using mediation, we tested whether FtF contact (M) mediated the effects of proximity of a relative (X) on social isolation (Y1) and loneliness (Y2) after controlling for age, gender, education, marital status, and area deprivation. Building on the two mediation models, we tested separately the moderating effects of telephone contact (W1), text/e-mail contact (W2), and video contact (W3) on the “b” paths. We used *PROCESS* (version 3.4), a computational procedure for SPSS (version 26), to implement mediation and moderated-mediation analysis (Hayes, 2017). Bootstrapped (5,000 random resamples) estimates of 95% confidence intervals (CI) were used to determine significant mediation. Significant moderation effects were ascertained by examining the index of moderation (an interval estimate of the parameter of a function linking the indirect effect to values of a moderator). The highest order of unconditional interaction (the specific interaction effect on the “b” path), and model change were also used to interpret effects, if these conflicted with the index of moderation. Effects of significant moderation were interpreted through graphing conditional effect at specific levels of the moderator.

## Results

### Descriptive Statistics

Supplementary Table 1 provides an overview of the descriptive statistics for the full sample *N* = 2,099 (*M*_age_*=* 75.99, *SD*_age_ = 6.54, *M*_education_ = 11.87, *SD*_education_ = 2.76, 49% male, 63% married) and analysis of sample characteristics for subsamples representing the younger *n =* 1,051 (*M*_age_ = 70.67, *SD*_age_ = 1.99, *M*_education_ = 12.71, *SD*_education_ = 2.81, 49% male, 74% married) and older *n* = 1,048 (*M*_age_ = 81.32, *SD*_age_ = .98 *M*_education_ = 11.57, *SD*_education_ = 2.67, *n* = 48% male, 52% married) cohorts. There were no significant differences in gender between younger and older cohorts, χ ^2^ (1, 2099) = 0.30, *p* = .586. However, a significantly larger proportion of the younger cohort were married χ ^2^ (1, 2099) = 109.09, *p* < .001 and had more education *t*(2097) = −5.02, *p* < .001 than the older cohort. Most participants lived in areas ranked in the third quintile of disadvantage.

More than one fifth (*n =* 465, 22.2%) of the sample were identified as isolated, scoring below 12 on the LSNS. Proportionally fewer participants in the younger cohort were isolated compared to the older cohort, χ ^2^ (1, 2099) = 14.99, *p* < .001. One quarter of the sample (*n =* 524, 25%) scored in the range of 2–6 on the loneliness scale, identifying this proportion of the sample as lonely (De Jong Gierveld & Van Tilburg, 2006). Proportionally fewer participants in the younger cohort were lonely compared to the older cohort χ ^2^ (1, 2099) = 10.01, *p* = .002.

On average, participants lived between 6 and 15 miles of at least one relative, and had FtF contact and phone contact with a relative at least weekly. Contact using other forms of technology-mediated communication was less frequent than FtF or phone contact; on average, participants were in contact with a relative by text/e-mail and video call less than monthly. There were significant differences between cohorts, with younger cohorts more frequently using these forms of technology-mediated communication than older cohorts: e-mail/text contact, *U* = 397,908.5, *p* < .001, *r* = −.27; video contact, *U* = 506,109.5, *p* < .001, *r* = −.12.

Bivariate correlation showed that the proximity of a relative was significantly associated with three covariates (marital status, education, and area disadvantage), the proposed mediator (FtF contact), all three proposed moderators (contact by phone, e-mail/text, and video), and both dependent variables (isolation and loneliness; Supplementary Table 2). Living closer to a relative was associated with being unmarried, fewer years of education, and living in an area of greater disadvantage. Overall, living further away from a relative was associated with poorer outcomes, that is, greater isolation (H1a) and loneliness (H2a). The VIF value for all predictors was <3, indicating that there was not a high degree of multicollinearity (Hair et al., 1995).

### Mediation Analysis

Figure 1B and C shows the effect of proximity to a relative on the mediator (“a” path) and the mediator’s effect on isolation (Y1) and loneliness (Y2) (“b” path), partialing out the effect of proximity to a relative (and correcting for control variables). The total effect (the sum of the direct and indirect effects) of proximity of nearest relative on isolation is significant (*c* = −.61, *p* < .001), but the direct effect on isolation is not (*c*^'^ = .147, *p* = .181). Similarly, the total effect of proximity of nearest relative on loneliness is significant (*c* = .11, *p* < .001), but the direct effect on loneliness (*c*^'^ = .02, *p* = .528) is not. Proximity to nearest relative has a significant indirect effect on social isolation (a^   b^ = −.76; 95% CI [−.93, −.58]) (H1b) and loneliness (a^   b^ = .10; 95% CI [.06, .14]) (H2b) through the mediating variable FtF contact.

### Moderated-Mediation Analysis

**Figure 2.**
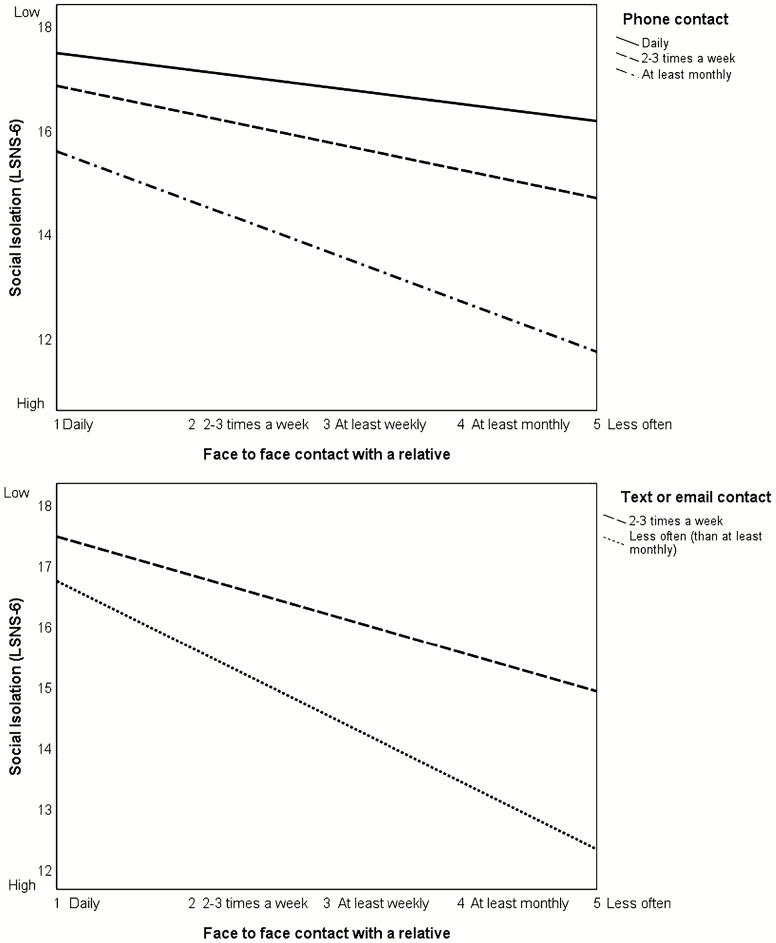
Graphs of the association between face-to-face contact and social isolation moderated by phone contact and text/e-mail contact for the total sample. Different values of phone contact represent 16th, 50th, and 84th percentiles. Different values of text/e-mail contact represent 16th and 50th percentiles (84th percentile is the same as the 50th).

The moderated-mediation models are summarized in Table 1. More frequent telephone contact was associated with less isolation. Phone contact also moderated the effect of FtF contact on isolation (H3a) 95% CI [−.24, −.08]. The bootstrapped conditional indirect effect of proximity of relative on isolation via FtF contact are summarized in Table 2 and show that at each value of the moderator, the negative effect of infrequent FtF contact on isolation is diminished. Figure 2 demonstrates that participants who were in phone contact with a relative at least monthly (but less than weekly), were more isolated than those who were in contact by phone more frequently. Isolation was particularly pronounced for infrequent phone users who also saw relatives FtF less than monthly. This group of older people could be classified as clinically isolated, on average scoring below 12 on the LSNS. On the other hand, daily contact with a relative by phone appears to protect against isolation, as participants who had daily phone contact with a relative alongside less than monthly FtF contact were less isolated than those participants who had daily FtF contact but only monthly phone contact.

**Table 1.** Coefficients for Moderated-Mediation Models with Social Isolation (Y1) and Loneliness (Y2) as Outcomes for the Total Sample (*N* = 2,099)

Outcome	Face-to-face contact (M)		Social isolation (Y1)		Social isolation (Y1)		Social isolation (Y1)	
Moderator			Phone contact (W1)		Text/e-mail contact (W2)		Video contact (W3)	
Predictor	Coeff. (*SE*)	*p*	Coeff. (*SE*)	*p*	Coeff. (*SE*)	*p*	Coeff. (*SE*)	*p*
Constant	.450 (.283)	.112	21.974 (1.472)	.000	19.900 (1.538)	.000	22.622 (1.935)	
Proximity to relative (X)	.736 (.013)	.000	.062 (.107)	.561	.073 (.109)	.506	.126 (.110)	.251
Face-to-face contact (M)	—		−.113 (.188)	.546	−.326 (.246)	.185	−.902 (.443)	.251
W	—		−.415 (.170)	.015	−.088 (.140)	.553	−.294 (.275)	.284
M × W	—		−.212 (.054)	.000	−.155 (.052)	.003	−.024 (.090)	.789
Age (C_1_)	.002 (.003)	.551	−.089 (.017)	.000	−.057 (.018)	.001	−.081 (0.17)	.000
Male (C_2_)	.163 (.042)	.000	−.557 (.220)	.011	−.838 (.223)	.000	−1.028 (.222)	.000
Married (C_3_)	−.018 (.046)	.704	.459 (.236)	.052	.508 (.241)	.035	.559 (.243)	.022
Education (C_4_)	.011 (.008)	.146	.168 (.039)	.000	.142 (.040)	.000	.160 (.040)	.000
Area disadvantage (C_5_)	.004 (.017)	.834	.269 (.087)	.002	.301 (.089)	.001	.311 (.090)	.001
			*R* ^2^ = .159 *F* (9, 2089) = 43.82, *p* < .001		*R* ^2^ = .123 *F* (9, 2089) = 32.68, *p* < .001		*R* ^2^ = .108 *F* (9, 2089) = 28.14, *p* < .001	
Outcome			Loneliness (Y2)		Loneliness (Y2)		Loneliness (Y2)	
Moderator			Phone contact (W1)		Text/e-mail contact (W2)		Video contact (W3)	
Constant			.169 (.351)	.631	.363 (.360)	.314	−.055 (.450)	.884
Proximity to relative (X)			.023 (.025)	.363	.024 (.026)	.357	.017 (.026)	.496
Face-to-face contact (M)			.036 (.045)	.427	.043 (.058)	.451	.183 (.103)	.075
W			.014 (.041)	.725	−.005 (.035)	.879	.056 (.064)	.383
M × W			.025 (.013)	.059	.020 (.012)	.110	−.011 (0.21)	.585
Age (C_1_)			.011 (.004)	.006	.008 (.044)	.055	.010 (.004)	.012
Male (C_2_)			.057 (.052)	.275	.079 (.052)	.130	.096 (.052)	.063
Married (C_3_)			−.298 (.056)	.000	−.303 (.056)	.000	−.310 (.057)	.000
Education (C_4_)			−.011 (.009)	.241	−.009 (.009)	.363	−.011 (.009)	.247
Area disadvantage (C_5_)			−.037 (.021)	.076	−.040 (.021)	.055	−.041 (.021)	.052
	*R* ^2^ = .613 F (6, 2092) = 551.57, *p* < .001		*R* ^2^ = .064 F (9, 2089) = 15.97, * p* < .001		*R* ^2^ = .060 F (9, 2089) = 14.85, *p* < .001		*R* ^2^ = .057 F (9, 2089) = 14.05, *p* < .001	

*Note:* Labels within the table reflect the following: X independent variable (proximity to relative), Y dependent variables (Y1 Social Isolation; Y2 Loneliness), M mediator variable (face-to-face contact), W moderator variables (W1 phone, W2 text/e-mail, W3 Skype), C_x_ covariates (C_1_ Age, C_2_ Male, C_3_ Married, C_4_ Education, C_5_ Area Disadvantage).

**Table 2.** Bootstrapped Conditional Indirect Effects of Proximity of Relatives on Social Isolation via Face-to-Face Contact at Specific Values of Moderators for Significant Moderation Models Only

	ModeratorPercentiles	Total sample			<75 years			≥75 years	
Mediator		Value	Coeff.	[Boot 95% CI]	Value	Coeff.	[Boot 95% CI]	Value	Coeff.	[Boot 95% CI]
	Phone contact (W1)									
	16th	1	−.239	[−.459, −.028]	1	−.421	[−.730, −.110]	1	−.102	[−.404, .199]
Face-to-face contact (M)	50th	2	−.395	[−.574, −.219]	2	−.572	[−.826, −.313]	2	−.263	[−.515, −0.11]
	84th	4	−.707	[−.913, −.505]	4	−.876	[−1.179, −.570]	4	−.585	[−.872, −.300]
	Text/e-mail contact (W2)									
	16th	2	−.468	[−.693, −.239]	2	−.706	[−.997, −.408]	3	−.366	[−.655, −.075]
	50th	5	−.811	[−.993, −.627]	4	−.916	[−1.158, −.669]	5	−.653	[−.896, −.408]
	84th	*			5	−1.021	[−1.293, −.746]	*		

*Note*: Labels within the table reflect the following: M mediator variable (face-to-face contact), W moderator variables (W1 phone, W2 text/e-mail).

*Eighty-fourth percentile is the same as 50th percentile. Five thousand bootstrap samples for percentile confidence intervals (CI).

Text or e-mail contact also moderated the effect of FtF contact on isolation (H3b) 95% CI [−.19, −.04] so that the negative effect of infrequent FtF contact on isolation is diminished. Graphing the moderation effects in Figure 2 demonstrates that older adults who were in text or e-mail contact with a relative more frequently (two to three times a week) reported less isolation in association with less frequent FtF contact with a relative, in comparison to those who texted or e-mailed less frequently (less often than monthly), even with infrequent FtF contact.

Video contact did not exert any direct or moderating effect on social isolation (H3c) 95% CI [−.15, .10]. None of the proposed moderators had a moderating effect on loneliness (H4a–c): phone contact 95% CI [−.00, .04], text/e-mail contact 95% CI [−.00, .03], and video contact 95% CI [−.04, .02].

### Group Comparisons of the Moderating Effects of Technology-Mediated Communication

First, we established that proximity to nearest relative had a significant indirect effect on social isolation (<75 years: a^   b^ = −.97; 95% CI [−1.23, −.73], ≥ 75 years a^   b^ = −.76; 95% CI [−.83, −.35]) and loneliness (<75 years: a^   b^ = .10; 95% CI [.03, .16], ≥ 75 years: a^   b^ = .09; 95% CI [.04, .15]) through the mediating variable FtF contact for both cohorts. Next, we ran six moderated-mediation models for each cohort (i.e., one model for each moderator [W1–W3] with each outcome [Y1, Y2]).

As demonstrated in the analysis of the full sample, we found that phone contact moderated the effect of FtF contact on isolation in both cohorts (H5a): < 75 years 95% CI [−.27, −.03]; ≥ 75 years 95% CI [−.27, −.06]. Text/e-mail contact also moderated the effect of FtF contact on isolation in both cohorts, not supporting our hypothesis (H5b): < 75 years 95% CI [−.21, −.00], ≥ 75 years 95% CI [−.27, −.03]. As for the full sample, video contact showed no significant moderation effect in either cohort (H5c): < 75 years 95% CI [−.17, .14], ≥ 75 years 95% CI [−.26, .17]. Table 2 shows that at each value of phone (W1) and text/e-mail (W2) contact, the negative effect of infrequent FtF contact on isolation is diminished by frequency of phone calls or text/e-mails with a relative (except for daily use of the phone (1) in the older cohort). In each model, the effects are stronger for the younger cohort.

**Figure 3.**
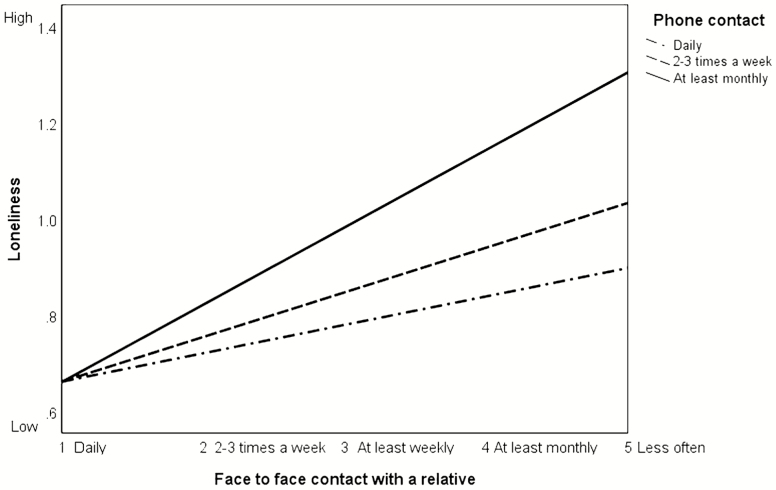
Graph of the association between face-to-face contact and loneliness moderated by phone contact for the sample < 75 years. Different values of phone contact represent 16th, 50th, and 84th percentiles.

Although we did not find any moderating effects in the loneliness models for the full sample, we observed one significant effect in the younger sample, albeit a weak effect. In this respect, at the highest order of unconditional interaction, phone contact moderated the effect of FtF contact on loneliness in the younger cohort 95% CI [.00, .07] (i.e., there was a significant effect on the “b” path). Moreover, including the interaction effect significantly changed the model *F*(1, 1040) = 3.49, *p* = .049. However, the conditional effects were only significant at two levels of the moderator (phone contact two to three times a week 95% CI [.04, .13] and at least monthly 95% CI [.04, .19]). Consequently, the index of moderated mediation was not significant, 95% CI [−.00, .05]. Telephone contact had no moderating effect on the “b” path for the loneliness model in the older cohort 95% CI [−.02, 04]; thus, we were unable to support our hypothesis that telephone contact would have emotional equivalence to FtF contact in both cohorts (H6a). Graphing the interaction effects in Figure 3 highlights that contact with relatives by phone two to three times a week reduces loneliness when FtF contact is less than daily. Moderation appears to be strongest for infrequent FtF contact; with daily FtF contact, the effect is similar across different levels of phone contact.

Text/e-mail and video contact did not have a significant moderating effect in the loneliness models for either cohort; thus, our other group comparison hypotheses were rejected (H6b, H6c).

## Discussion

Widespread economic, political, and technological transformations brought about by globalization have had an impact on the dispersion and communications practices of families and older people. Our analyses have demonstrated that family proximity impacts on social isolation and loneliness in later life. The further away relatives live, the less frequently an older person is likely to see kin, and the more likely they are to experience greater isolation or loneliness.

There has been a revolution in technology and communication that has influenced older people and family relations. Technology-mediated communication has been promulgated as a tool that can be used by geographically dispersed families to defend against isolation and loneliness of remotely located older kin. Certain demographic characteristics of the participants influenced technology-mediated communication with relatives. Greater age, being unmarried, having fewer years of education and living in a disadvantaged area were associated with less frequent text/e-mail and video contact. In contrast, women used the phone or text/e-mail more frequently than men. While these characteristics influenced the use of technology (and outcomes), the statistical models corrected for the potential confounders and estimated the paths in an unbiased way. This study sought to establish what functional and emotional equivalence with FtF contact these forms of technology have within the family relationships of older people living in Wales, UK. In this respect, embodied, copresent FtF contact is conceptualized as the norm against which other kinds of communication are compared (Baym, 2015).

Our research moved beyond experimental research strategies (i.e., intervention studies). Some studies have demonstrated that delivering therapies via technology-mediated communication (e.g., telepsychiatry) positively impacts on depression, general mental health, and wellbeing (Kaonga & Morgan, 2019). However, these interventions are rarely targeted specifically at older people and do not address loneliness per se. In a meta-analysis of the effectiveness of loneliness interventions (Masi, Chen, Hawkley, & Cacioppo, 2010), there were only 10 studies conducted with older people using technology-mediated communication. Many of these studies were conducted with very specific groups of older people [e.g., who called a suicide help line (Morrow-Howell et al., 1998)] and only two demonstrated a significant reduction in loneliness [telephone social support to older blind veterans in the United States (Evans & Jaureguy, 1982) and computer training to increase social opportunities for older Israelis (Shapira et al., 2007)]. Very few robust studies have examined the everyday communication practices of older people and the impact on loneliness.

We focused on “naturally occurring” contexts in which technology-mediated communication was already being used. We examined older people’s key practices of everyday life “doing family” and found that synchronous audio and asynchronous written forms of communication—phone and text/e-mail contact—both have some functional equivalence with FtF contact. Telephone calls, text, and e-mail are used as a substitute for or to supplement in-person contact and reduced the influence of FtF contact on social isolation for older people in both age cohorts. Video calls were not frequently used, and did not have an influence on social isolation when taking into account proximity and FtF contact with family members. The “oldest” technology (telephone, text, and e-mail) are now part of routine communication practices for older people, but the more recent technology—video calls—are used less frequently.

The analyses focused on older people’s familial relationships—a special relationship in which both parties already know one another. We found that none of the technology-mediated communication moderated the impact of FtF contact on loneliness for the full sample. In this respect, none of the forms of communication have emotional equivalence to the “gold standard” of embodied presence. Our interpretation of these results is that the contact provided through technology-mediated communication does not match up to older people’s expectations concerning family relationships in later life.

We contrasted the moderated-mediation loneliness models for cohorts of older people, believing that it would be more likely that the younger cohort had greater familiarity with the technology used for text/e-mail and video contact. We thought that more familiarity with this technology would influence expectations for social interaction (Baym, 2015), decreasing the likelihood of a mismatch between achieved and desired social relations and loneliness. However, we found limited evidence that telephone contact (but not e-mail/text and video contact) had a moderating influence on loneliness for the younger cohort only. Although the results did not support our hypotheses, they lend some credence to our underlying theoretical reasoning, and provide some evidence that phone contact may have emotional equivalence to FtF contact in the younger cohort.

The weak moderating influence of telephone contact on loneliness observed in the younger cohort could suggest there is a normative lag between the functional and emotional equivalence of communication technologies to FtF contact; that is, there is a delay between widespread use of technology-mediated communication in familial contact and incorporating it into expectations concerning familial relationships. In the future, technology-mediated communication may impact on loneliness if expectations “catch up” with the reality of contact. However, expectations for familial relations are dependent on other factors not captured in our analytical models, such as media representations and/or life-course experiences of technology-mediated communication (Burholt et al., 2019). Consequently, the manner in which technology-mediated communication is socially constructed as having utopian or dystopian consequences for relationships, and/or the ways in which they compare experientially with in-person embodied contact, may result in enduring social values that rate technology-mediated communication as inferior to FtF contact.

The analytical models in this study are only a partial representation of social isolation and loneliness in the older population. Firstly, the research outlined in this article was conducted in Wales, UK, and the models should be tested with data from other countries to ascertain the applicability in other cultural contexts where the use of technology-mediated communication and expectations about familial relations may differ. Secondly, questions about technology-mediated communication were only asked in Wave 2 of the study. Although CFAS Wales was a panel cohort study the cross-sectional nature of data means that we cannot be sure of the direction of causality. Thirdly, our sample comprised older people with living relatives, and we did not include variables that represented frequency or quality of contact with friends. Further analysis is warranted to test the effects of technology-mediated communication on isolation and loneliness in nonkin relationships and within older people’s networks of social relations. Fourthly, this analysis relied on our interpretation of data on technology-mediated communication and its functional and emotional equivalence to FtF contact, rather than capturing the meaning of human action. While loneliness and isolation are quantifiable phenomena, qualitative data could provide a deeper insight into older people’s emotional responses to connecting with their families through technology. Fifthly, mediation and moderated-mediation models provide only two examples of pathways to loneliness, and other models may fit the data better. For example, we have included only one moderator in each model. Future research may consider whether the cumulative effect of polymedia [the use of a wide variety of communication media (Madianou & Miller, 2012)] in communication is important. Additionally, the models might be improved by including other variables such as functional, visual, and auditory capacity alongside communication preferences which are likely to impact on functional and emotional equivalence to FtF contact.

Different forms of technology-mediated communication can either enable or disable elements that contribute to emotional recognition, such as facial expression, direction of gaze, and voice intonation (Baym, 2015). Text and e-mail are asynchronous and have “cues filtered out” (Walther & Parks, 2002). Consequently, telephone calls may be difficult for people with cognitive or auditory impairment who may prefer visual cues, whereas texting or sending e-mails may be difficult for people with chronic pain or hand impairments (Moffatt et al., 2013). Alternatively, people with limited mobility may be predisposed to using technology-mediated communication more frequently, and/or may have altered their expectations about the means by which they can sustain family relationships. Personal abilities and preferences for forms of communication are likely to impact both on use (functional equivalence to FtF contact) and meeting expectations for contact with family members (emotional equivalence to FtF contact). CFAS Wales did not include any questions about communication preferences, but future studies could include Likert-type scales that could be used to model effects.

In conclusion, our study has shown that social isolation can be precipitated by distance from kin and mediated by low frequency of FtF contact; the effect can be diminished by telephone, text, and e-mail contact (but not video contact). While loneliness can also be predicted by distance from kin, and mediated by low frequency of FtF, it is not moderated by technology-mediated communication. In our interpretation of the statistical models, we have posited that the influence of technology-mediated communication on outcomes may change over time depending on personal resources, preferences, and experiences of technology-mediated communication, alongside culturally informed perceptions of the value of technology-mediated communication in familial relationships. The study demonstrates the importance of theorizing about the pathways to isolation and loneliness, as this knowledge can be used to better understand the likelihood of implementing successful interventions and the impact of current technological trends on the older population.

Interventions that fall under the rubric of improving opportunities for social connection often seek to decrease isolation and loneliness by providing technology-mediated communication resources or training to older people. While interventions in this category have shown some impact on loneliness (e.g., Shapira, Barak, & Gal, 2007), the role of technology-mediated communication in sustaining familial relationships has not been ascertained. Moving beyond the experimental setting, this article has demonstrated that although using telephone calls, texts, and e-mail to connect to family members may exert an influence on social isolation, it does not have a strong impact on loneliness. In the real world, despite the proliferation of smart phone ownership, there is variation by age group (Rossiter-Base, 2019) and our analytical models also suggest that technology-mediated communication are unlikely to have an equal impact on isolation and loneliness across age cohorts. Loneliness is influenced by norms and life-course experiences, and unless the quality and quantity of family contact provided by technology-mediated communications meets the expectations of older people, it will not be effectively reduced. In the long run, research that takes into account variations in use, preferences, and normative values between cohorts or other subgroups of older people could inform interventions tailored to meet the multifarious pathways to isolation and loneliness.

## Supplementary Material

gnz179_suppl_Supplementary_Material
