# Supplementary material for: Technology-Mediated Communication in Familial Relationships: Moderated-Mediation Models of Isolation and Loneliness
Source: Gerontologist. 2020 May 5;60(7):1202–12. doi: 10.1093/geront/gnaa040 (PMC8059137; doi:10.1093/geront/gnaa040)
Supplement: gnz179_suppl_Supplementary_Material [file gnz179_suppl_supplementary_material.docx]

**Supplementary materials**

**Covariates in the Mediation and Moderated-Mediation Analysis**

In the mediation models, the covariates representing age, gender, marital status education, and area deprivation were significantly associated with either the mediation variable (FtF contact) or the dependent variables (social isolation and loneliness). Greater age, and being unmarried were associated with greater social isolation and loneliness. Being male was associated with greater social isolation, and less frequent FtF contact with a relative. Although there was a significant association between more years of education and less isolation, living in the least deprived area was associated with more social isolation (Figure 1B and 1C).

The same significant effects were observed in the moderated-mediation models, with the exception of one: marital status was not significantly associated with social isolation in the model incorporating telephone contact as a moderator.

Supplementary Table 1. Descriptive Statistics for the Sample (*N*=2099) and Sub-Samples for Younger (*n*=1051*)* and Older *(n*=1048*)* Cohorts

|  | <75 years | ≥75 years | All | *p* |
| --- | --- | --- | --- | --- |
| Age, mean (SD) years ^A^ | 70.67 | 81.32 | 75.99 (6.54) | *** |
| Male % ^B^ | 49 | 48 | 49 |  |
| Married % ^B^ | 74 | 52 | 63 | *** |
| Education, mean (SD) years ^A^ | 12.17 (2.81) | 11.57 (2.67) | 11.87 (2.76) | *** |
| Area Disadvantage, median (SD) quintile ^C^ | 3 (1.22) | 3 (1.25) | 3 (1.24) |  |
| Proximity of relative, median (SD) ^C^ | 2 (1.66) | 2 (1.55) | 2 (1.57) |  |
| FtF contact, median (SD) ^C^ | 2 (1.50) | 2 (1.49) | 2 (1.49) |  |
| Social isolation, mean (SD) ^A^ | 15.99 (5.14) | 14.86 (5.11) | 15.42 (5.16) | *** |
| Loneliness, mean (SD) ^A^ | 0.86 (1.09) | 1.05 (1.23) | 0.95 (1.17) | *** |
| Phone contact, median (SD) ^C^ | 2 (1.24) | 2 (1.26) | 2 (1.25) |  |
| Email/text contact, median (SD) ^C^ | 4 (1.54) | 5 (1.23) | 5 (1.44) | *** |
| Video contact, median (SD) ^C^ | 5 (0.88) | 5 (0.70) | 5 (0.80) | *** |

*Notes*: Group differences were tested using ^A^ t tests for continuous variables, ^B^ chi-square statistics for categorical variables and ^C^ Mann Whitney U tests for ordinal data.

*p < .05. **p < .01. ***p < .001.

Supplementary Table 2. Correlation Analysis for the Full Sample (*N*=2099)

|  |  | 2 | 3 | 4 | 5 | 6 | 7 | 8 | 9 | 10 | 11 | 12 |
| --- | --- | --- | --- | --- | --- | --- | --- | --- | --- | --- | --- | --- |
| 1 | Age | -.05 | .29** | -.15** | .01 | -.01 | -.00 | -.13** | .08** | -.02 | .32** | .13** |
| 2 | Male |  | .26** | -.02 | .00 | .03 | .07** | -.13** | .03 | .20** | .13** | .02 |
| 3 | Married |  |  | .10** | .12** | .06* | .06** | .07** | -.12** | .05* | -.11** | -.09** |
| 4 | Education (years) |  |  |  | .22** | .16** | .15** | .10** | -.03 | .05* | -.16** | -.10** |
| 5 | Area disadvantage |  |  |  |  | .17** | .14** | .06** | -.01 | .03 | -.08** | -.06** |
| 6 | Proximity of relative |  |  |  |  |  | .76** | -.16** | .13** | .21** | -.09** | -.12** |
| 7 | FtF contact with relative | |  |  |  |  |  | -.22** | .15** | .34** | -.04 | -.08** |
| 8 | Social isolation |  |  |  |  |  |  |  | -.27** | -.26** | -.19** | -.10** |
| 9 | Loneliness |  |  |  |  |  |  |  |  | .11** | .06* | .02 |
| 10 | Phone contact |  |  |  |  |  |  |  |  |  | .11** | .06* |
| 11 | Text/email contact |  |  |  |  |  |  |  |  |  |  | .26** |
| 12 | Video contact |  |  |  |  |  |  |  |  |  |  |  |

* *p<*.05 ** *p*<.01
